# Evaluation of trabecular bone in individuals with periodontitis using fractal analysis: An observational cross-sectional study

**DOI:** 10.4317/jced.60921

**Published:** 2023-12-01

**Authors:** Fatma-Ucan Yarkac, Melek Tassoker, Ummuhan-Tekin Atay, Duygu Azman

**Affiliations:** 1 Necmettin Erbakan University, Faculty of Dentistry, Periodontology, Konya, Turkey; 2 Necmettin Erbakan University, Faculty of Dentistry, Oral Diagnosis and Radiology, Konya, Turkey

## Abstract

**Background:**

It is very difficult to objectively evaluate the negative changes in bone structure due to periodontitis. The present study was aimed to evaluate the trabecular bone structure between healthy individuals and periodontitis patients by fractal analysis (FA) on digital panoramic radiographs.

**Material and Methods:**

The study included 50 periodontally healthy individuals (control group), 50 individuals with Stage 1 periodontitis (S1-P group), 50 individuals with Stage 2 periodontitis (S2-P), and 50 individuals with Stage 3 periodontitis (S3-P), a total of 200 individuals were included. The fractal dimension (FD) value of the trabecular bone in the interdental space between mandibular first molar and second premolar tooth roots was evaluated using Image J program. The mean FD values of the two regions were calculated by box counting method.

**Results:**

There was a statistically significant difference between the groups in terms of all periodontal parameter values (*p*<0.05). The mean FD values of individuals diagnosed with periodontitis were 1.36±0.08 in the S1-P group, 1.35±0.07 in the S2-P group, 1.28±0.15 in the S3-P group, and 1.44±0.06 in the control group. When the FD values between the groups were examined, it was seen that there was a statistically significant difference between the control and individuals with periodontitis, and the mean FD values were significantly higher in the healthy group (*p*<0.05). The best receiver operator curve was identified for periodontitis at the ≤1.409 cut-off FD value (area under the curve: 0.828; 95% CI: 0.758-0.899); *p*=0.000, *p*<0.001).

**Conclusions:**

FD evaluation can give an objective result about the effect of periodontitis on alveolar bone. The FD values of trabecular bone are different in healthy individuals and individuals with different stages of periodontitis. The findings suggested that a negative correlation between the periodontal data with the sites in which FD was measured and as the periodontitis stage progresses, FD decreases.

** Key words:**Diagnosis, Periodontal Diseases, Radiographic Evaluation.

## Introduction

Periodontitis is a chronic inflammatory disease caused by specific microorganisms, affecting the supporting tissues of the tooth, in which inflammation of the gingiva, pocket formation, clinical attachment and alveolar bone loss occurs (1). Accurate determination of bone loss in the alveolar bone is important for the diagnosis, treatment and prognosis of the disease (2). Although radiography is one of the most suiTable methods for periodontal diagnosis, it has been shown to be of limited value for the early detection of subtle bone changes (3). Conventional radiographs can reflect changes in bone following a 30% to 50% resorption of the bone structure (4).

Therefore, further radiographical evaluation methods such as fractal analysis (FA) have been recommended for detailed analysis of changes in alveolar bone in periodontitis (5). It has been stated that fractal analysis may be useful in detecting small trabecular changes before bone loss progresses and in stopping the progression of the existing disease, since periodontitis causes a decrease in the amount of bone and deterioration in trabecular integrity (6).

The term fractal is derived from the Latin word ‘fractus’ meaning fragmented or broken and is used to describe curves, unrelated scattered points, surfaces and amorphous structures that have no examples in standard geometry (7). The complexity of shapes or objects with fractal branching is calculated by fractal analysis, and its numerical value is expressed as fractal dimension (FD). FD represents the complexity of the structure by measuring its similarity to itself (8).

FA is becoming an increasingly common practice in the evaluation of bone structure. It is stated that FD detected on radiographs reflects the changes in trabecular bone density and mineral loss in the bone. Since trabecular bone has a higher metabolic activity than cortical bone, it is more decisive in the evaluation of changes in bone structure (9,10). A low value of FD indicates a higher proportion of cavities in the bone and a more porous structure of bone tissue, whereas a high value of FD shows that the bone structure is more complex, denser and the spaces in the bone are less (7,11).

The FA method has a wide range of uses because it is non-invasive, easily accessible and not affected by variables such as projection geometry. It has been used in many different areas of dentistry; such as monitoring the healing of periapical lesions after root canal treatment, evaluation of the prognosis of orthognathic surgery cases and primary stability in implant treatments, dental material analysis and the diagnosis of temporomandibular joint disorders, caries and periodontal diseases (11).

It is very difficult to detect changes in the alveolar bone due to periodontitis with clinical findings and conventional radiographs (12). In the literature, alterations in trabecular bone were examined by FA method in patients with periodontitis, and it was reported that there were significant differences in the fractal values of alveolar bone between individuals with periodontitis and control groups (5,13). However, few studies have individually evaluated periodontitis as mild, moderate, and severe (14).

Therefore, the aim of this study is to evaluate trabecular bone structure in the panoramic radiographs of patients with different stages of periodontitis using FA.

## Material and Methods

-Study sample

The present study was carried out with the participation of individuals who applied to the periodontology clinic at the Necmettin Erbakan University Faculty of Dentistry for treatment and volunteered to be included in the research. This observational cross-sectional study (NCT05639582) was conducted in accordance with the Helsinki Declaration and was approved by the Ethics Committee of Necmettin Erbakan University Faculty of Dentistry (Decision No: 2020/02-11). All participants provided a written informed consent to participate in this study.

The individuals included in the study were informed in detail about the study before the examination and an ‘Informed Consent Form’ was signed by all individuals who volunteered to participate in the study. Individuals with any systemic disease, any jaw pathologies, those using drugs that may affect bone metabolism, radiographs with poor diagnostic quality, and teeth with root canal treatment or periapical lesions on the region of interest (ROI), and individuals who had fractures or trauma affecting the anatomy or integrity of mandible were excluded from the study. Based on clinical and radiographic findings, a total of 200 periodontally healthy individuals and patients diagnosed with stage 1, 2 and 3 periodontitis were randomly selected and included in the study.

-Clinical examination

All clinical measurements (plaque index (PI), gingival index (GI) (15), probing pocket depth (PPD), and clinical attachment loss (CAL)) were performed by a calibrated physician (U.T.A.) using a Williams periodontal probe (Hu-Friedy, Chicago, Illinois, USA). USA) from 6 regions of each tooth (mesio-buccal, middle buccal, disto-buccal, mesio-lingual, middle lingual, and disto-lingual) (15). All teeth, excluding third molars, were examined. PPD is the distance between the bottom of the periodontal pocket and the free gingival margin, while CAL is defined as the distance between the bottom of the periodontal pocket and the cemento-enamel junctions. A millimetrically calibrated is used by periodontal probe for these measurements.

Ten randomly selected patients that were not included in the study were evaluated to estimate the reliability of the clinical measurements before the study. The intra-class correlation coefficient (ICC) was 0.96±0.03 for probing pocket depths measurements.

The patients were categorized by their periodontal health status, according to the 2017 classification (16) into four categories: Periodontally healthy (control), stage I (S1-P), stage II (S2-P), stage III (S1-P) periodontitis. Patients with a clinical attachment loss of 1-2 mm were grouped as stage I periodontitis (S1-P), individuals with 3-4 mm as stage II periodontitis (S2-P), and individuals with more than 4 mm as stage III periodontitis (S3-P). Patients with stage 4 periodontitis were not included in the study. Participants included in the periodontitis group had not received periodontal treatment before. In addition, patients with no radiographic bone loss or clinical attachment loss, having a bleeding on probing <10% and probing pocket depth<3mm, were diagnosed as periodontally health (control).

-Image acquisition

All panoramic radiographs within the scope of the research were obtained by 2D Veraviewpocs (J MORITA MFG corp, Kyoto, Japan) digital panoramic x-ray device in line with 70 kVp, 5 mA and 15 sec radiation parameters. Panoramic radiographs were standardized by positioning the Frankfort plane parallel to the horizontal plane and the midsagittal plane perpendicular to the horizontal plane. A 2.66 GHz Intel Xeon PC with 3.25 Gb RAM, Windows XPTM Professional operating system and a 27-inch flat panel color screen (Dell U2711HTM) with a resolution of 2,560×1,600 pixels were used for examining the radiographs.

-Image processing

In order to determine the intra-observer agreement, FD measurements were repeated in 20% of 200 patients (n=40) twice by the same observer (MT) with 11 years of oral radiology experience, with an interval of 14 days. Panoramic x-rays of the individuals included in the study were saved in the ‘TIF’ (Tagged Image File) format. For the standardization of radiographs, the dimensions of all images were adjusted to 2800×1500 pixels with the Adobe Photoshop CS5 (Adobe Systems Inc., San Jose, CA) program. For FA, ImageJ v1.52 program bundled with 64 Bit Java for Windows, which is a version of the National Institutes of Health Image software, was used. The program has been downloaded from the internet at https://imagej.nih.gov/ij/download.html.

**Figure 1:**
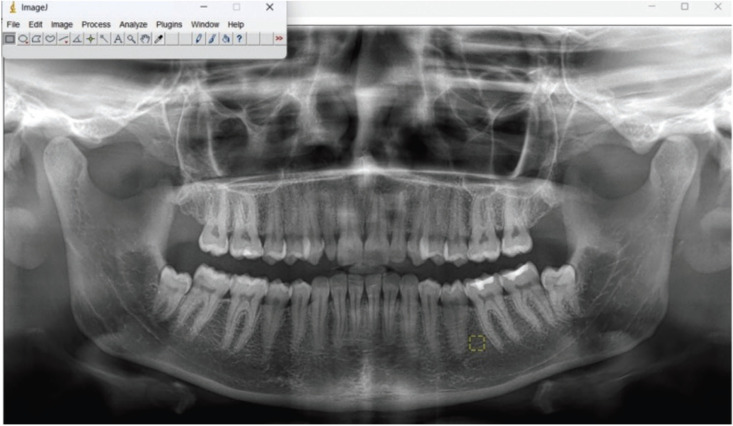
Demonstration of specified ROIs on the program.

Measurements were made on the ROI (Region of Interest) with a size of 50x50 pixels on the panoramic radiograph, from the area between the interdental regions of the mandibular second premolar and first molar teeth (not including the periodontium of the teeth and the cortical borders of the mandibular canal) (Fig. 1). Although bone loss in periodontal disease starts from the interproximal region, it may not be possible to select an ROI that is wide enough to analyze from the interdental region, especially in healthy individuals. For this reason, 50x50 pixel size ROIs were selected from the region as close to the interdental middle third as possible.

FA operations were performed using the box-counting method designed by White and Rudolph (17).

**Figure 2:**
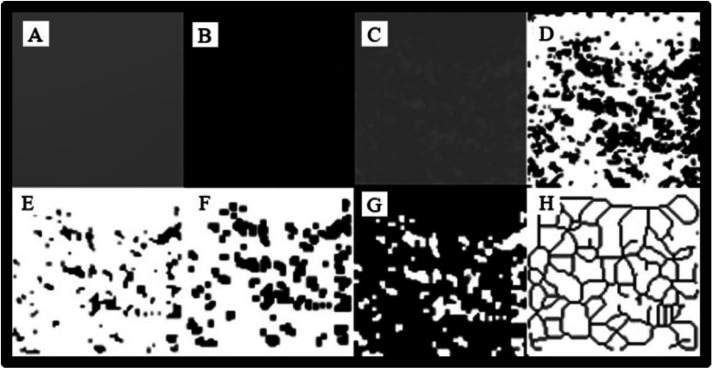
A, Blurring; B, Subtracting the blurred image from the original image; C, adding 128 shades of gray; D, Black-and-white image conversion; E, noise reduction with Erode; F, Expansion with Dilate; G, Color inversion; H, Conversion into skeletal format.

The image is blurred by applying a Gaussian filter (sigma, 35 pixels) to the duplicated image (Fig. 2A) then subtracted from the original image with the ‘subtraction’ process (Fig. 2B) and 128 gray values are added for each pixel (Fig. 2C). The resulting image is then converted into a black-and-white two-color format through the ‘Make Binary’ process (Fig. 2D). In order to reduce the noise on the image, the ‘Erode’ step is performed (Fig. 2E), followed by the ‘Dilate’ step to enlarge the existing areas and make the image more prominent (Fig. 2F). In the ‘invert’ process, the outlines of the trabecular bone are revealed by converting the white areas representing the trabecular bone on the image into black and the black areas representing the bone marrow into white (Fig. 2G). The inverted image is skeletonized by the ‘Skeletonize’ step, so that only the central parts of the trabeculae remain. Using the ‘box-counting’ function in the ImageJ program, the image is optimized to perform FD analysis on the skeletonized image (Fig. 2H).

For FD calculation, the image is divided into squares with dimensions of 2, 3, 4, 6, 8, 12, 16, 32, 64 pixels using the ‘Fractal box counter’ option beneath the ‘Analyze’ button. For pixels of different sizes, the squares containing trabeculae and the total number of squares in the image are calculated. These values are plotted on a logarithmic scale; the slope of the line that best fits 

the points on the graph gives the FD.

-Statistical Analysis

The sample size was calculated based on the alteration in FD levels (α = 0.05, effect size d = 0.49) as a primary outcome (18).At a power of 95% and significance level of 5%, it was determined that nearly 50 subjects per group were included in this study.

Data were evaluated with the SPSS 21.0 (IBM Corp, Armonk, NY, USA) program. Intra-class correlation coefficient (ICC) analysis was used to assess intra-observer reliability. Areas under the receiver operator characteristic curve (ROC) were studied to determine cut-off FD point for periodontitis. Descriptive statistics were calculated for all parameters in the study. The continuous numerical variables were tested for normal distribution by Kolmogorov-Smirnov test. Demographic data between groups, clinical periodontal parameters and FD values were evaluated with the One-way ANOVA test. Post hoc comparisons were made for the detected differences with One-way ANOVA test with Bonferoni corrections.

The relationship between FD and age was analyzed with the Pearson correlation coefficient. Independent sample-T test was used for analysis between gender and FD. Type-I error value was accepted as 5% in all analyzes and *p*<0.05 was considered statistically significant.

## Results

The ICC value, which tests the reliability of repeated measurements, is 0.862 (*p*=0.000), and values above 0.8 indicate good reliability (19). Two hundred subjects had panoramic radiographs available for analysis. Panoramic radiographs were taken from 50 individuals in the healthy (control) group (17 males and 33 females), 50 patients in S1-P group (14 males and 36 females), 50 patients in S2-P group (21 males and 29 females), and 50 patients in S3-P group (21 males and 29 females). Demographic information of the study participants is given in Table 1. There was no statistically significant difference in the distribution of the gender between the periodontitis patients and the healthy groups (*P* =0.392).

**Table 1:**
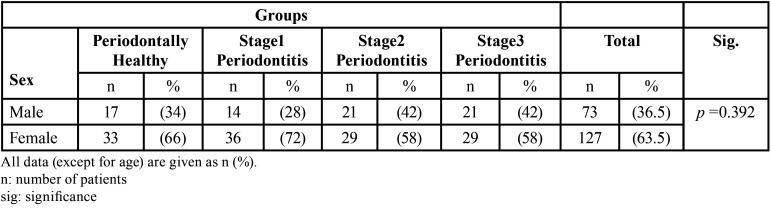
Distribution of healthy individuals and patients with peridontitis by gender.

The mean age of all four groups combined was 39 years; while the mean age was 30 years in the control (healthy) subgroup, 40 years in the mild periodontitis subgroup, 39 years in the moderate periodontitis subgroup and 45 years in the severe periodontitis subgroup (Table 2). The individuals in the healthy group were found to be significantly younger (*p*=0.005).

**Table 2:**
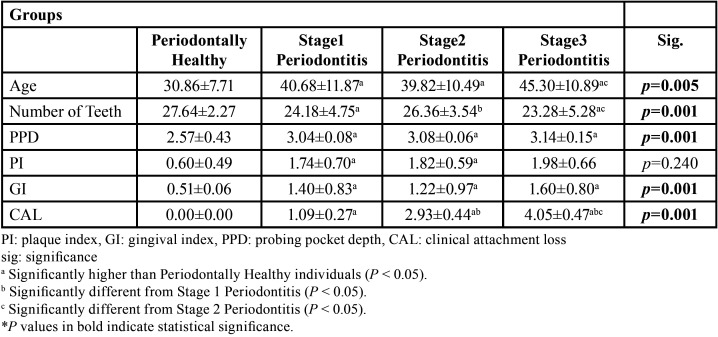
Clinical periodontal measurements (mean ± standard deviation).

The mean FD was 1.44 in the control (healthy) subgroup, 1.36 in the mild periodontitis subgroup (S1-P), 1.35 in the moderate periodontitis subgroup (S2-P), and 1.28 in the severe periodontitis subgroup (S3-P) (Table 3). When the FD values of all groups were examined, it was seen that there was a statistically significant difference between the healthy group and periodontitis groups, and the mean FD value was significantly higher in the healthy group (*p*=0.001).

**Table 3:**
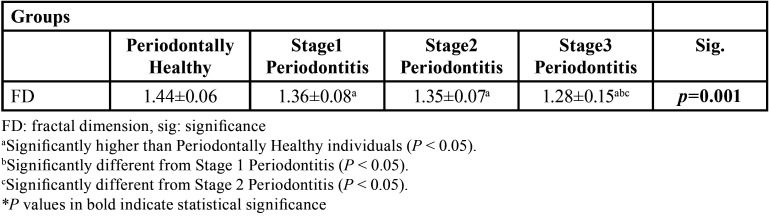
FD Values (mean ± standard deviation).

On the other hand, there was a statistically significant negative correlation was found between periodontal data with the sites in which FD was measured (*p*<0.001) (Table 4). In addition, a statistically significant negative correlation was found between periodontal parameters and age.

**Table 4:**
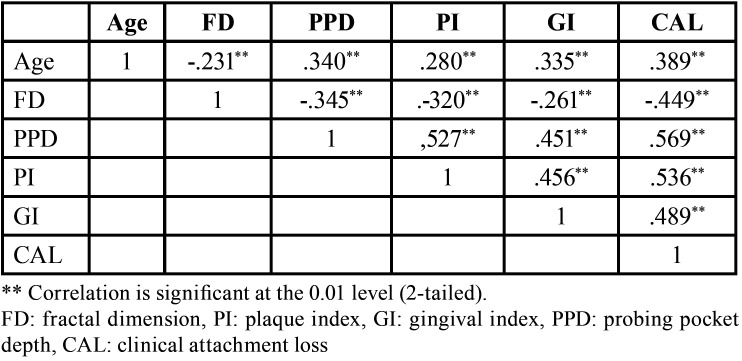
Summary of Intercorrelations for scores on FD, Periodontal Clinical Parameters and Age.

When receiver operating characteristic (ROC) analysis was performed, a FD value≤1.409 predicted the presence of periodontitis with a sensitivity of 74% and a specificity of 74% (area under the curve: 0.828; 95% CI: 0.758-0.899); *p*=0.000, *p*<0.001).

There was no statistically significant relationship between gender and FD (*p*=0.114). A statistically significant negative weak correlation was found between age and FD measurements (*p*=0.004, r=-0.231). FD was decreased with increasing age.

## Discussion

The results of the present study showed that FA using panoramic radiographic images can be successfully operated to differentiate healthy trabecular bone from trabecular bone with periodontitis at different stages (Stage I, II, III). Periodontitis causes loss of mass in the alveolar 

bone and deterioration of the trabecular structure (1). Image processing methods such as FA have been recommended for detailed analysis of changes in alveolar bone in periodontitis.Accurate determination of bone loss in the alveolar bone is important for the diagnosis, treatment and prognosis of the disease (5,14).

It is stated that the mandibular premolar region is the most ideal region for FA (20) and the mandible is mostly preferred in studies (21). Therefore, in this study, FD for the trabecular bone was calculated from the ROI determined in the mandibular premolar-molar region. Trabecular bone is preferred because it is metabolically more active than cortical bone (22).

A number of previous studies have been conducted on the application of fractal analysis in the detection of periodontitis based on radiographs (5,6,18,23). Shrout *et al*. reported that FD values of the interdental bone in the posterior mandibular region were higher in patients with gingivitis than in patients with periodontitis (5). Updike and Nowzari, in their study of the mandibular anterior teeth of healthy individuals and patients with moderate and severe periodontitis, concluded that FD values were higher in healthy periodontal bone (6). Soltani *et al*. examined patients diagnosed with periodontitis in 3 groups as mild, moderate and severe periodontitis and analyzed periapical radiographs and trabecular bone changes using FA. While they found a significant difference in FD values between the periodontally healthy group and the groups with moderate and severe periodontitis, they did not find a significant difference between the healthy group and the group with mild periodontitis (23). Cha *et al*. have reported that the FD values of patients with periodontitis are statistically significantly lower than those of healthy individuals, and that as the periodontal disease progresses, higher amounts of alveolar bone is lost and trabecular organization is disrupted (13). Sener *et al*. reported that trabecular changes can be quantitatively detected by FA in periodontally healthy individuals and patients diagnosed with moderately severe periodontitis (18). Similarly, in a study conducted by Aktuna Belgin *et al*. it was presented that alterations in the alveolar bone can be detected by FA using digital radiographs in patients with periodontitis (24). In light of the results of the FA performed with panoramic radiographic images used in the present study, it was observed that there were significant differences in FD values between periodontally healthy individuals and patients diagnosed with stage I, II and III periodontitis. Moreover, a statistically significant relationship was found between the periodontal status and FD values. The results of this study demonstrated that trabecular changes in the alveolar bone of patients with periodontitis of different severity can be detected with the aid of FA method and panoramic radiographs. Since the AUC according to the ROC analysis has a high value (0.828, *p*=0.000), it would be reasonable to consider FD=1.409 as the limit for periodontal health for the ROI selected from the premolar-molar region on panoramic radiography.

Differences in the size and shape of the jaw bones, medical history, and the distinct dynamics of bone metabolism may rule out gender-specific factors. While gender-related differences were reported in the iliac and vertebral cancellous bones in the literature, no significant differences were found in the jaw bones in other studies (18). Aktuna Belgin *et al*. and Updike *et al*. reported that there was no significant difference in the FD values of the jawbones according to gender (6,24).

In this study, similar to the literature, no statistically significant differences were found in the FD between genders.

The mean age of the periodontitis groups in this study was higher than the healthy group. When the correlation between age and FD measurements was examined, it was observed that FD decreased with increasing age. Since the severity of periodontal disease increases with advanced age, it should be considered that the decrease in FD is affected by age as well as periodontal disease. The lower mean age in healthy individuals may also have affected the findings. Since the frequency of periodontal healthy individuals decreases in advanced age, the age distribution in the data set used is the limitation of our study. There is a need for a study to be conducted with sample groups in which age and gender are matched in all groups.

Advanced age is a significant risk for osteoporosis. It has been reported that bone mineral density decreases in osteoporosis and FD decreases when bone density decreases (20). On the contrary, Yasar and Akgunlu reported that FD did not differ in osteoporosis and non-osteoporosis individuals. Differences such as sample distributions, systemic diseases of individuals, ROI selection, and imaging technique used can be thought to be effective in the difference in results (25).

## Conclusions

The results of this study showed that the FD values of trabecular bone are different in healthy individuals and individuals with different stages of periodontitis. FA can quantitatively and objectively detect alterations in the interdental trabecular pattern of patients with periodontitis. Measuring FD below 1.40 should suggest that periodontal disease affects trabeculation.
